# Anisotropy of the Drawing Plane in Focal Hand Dystonia: A Case Report and a Novel Postulate of Dysfunctional Tensorial Networks

**DOI:** 10.7759/cureus.13191

**Published:** 2021-02-07

**Authors:** Hassan Kesserwani

**Affiliations:** 1 Neurology, Flowers Medical Group, Dothan, USA

**Keywords:** focal dystonia, movement science / motor control

## Abstract

The pathophysiology of the dystonias has been associated with loss of inhibition in the sensory-motor cortex, brainstem and spinal cord, abnormal motor preparation in the pre-motor cortex, abnormal sensory processing in the sensory cortex, maladaptive plasticity and abnormal sensory-motor integration in the cerebral cortex. We present a case of focal hand dystonia with variance of spiral drawing in the horizontal and vertical planes, that is amelioration of spiral drawing in the vertical plane compared to the horizontal plane. We refer to this phenomenon as “anisotropy”. We seize upon this unique finding and postulate a novel mechanism for the generation of the dystonias. The anisotropy referred to can only be explained by a breakdown of the tensorial transformation of a covariant vector to a contravariant vector in the frequency hyperspace of the brain. This novel hypothesis is based on the unequivocal invariance of tensorial transformation, a mathematical fact in differential geometry, the very geometry of the most celebrated and sublime General Theory of Relativity, and the model of dynamic brain function proposed by Pellionisz and Llinas.

## Introduction

In order to understand the phenomenon of dystonia, we need to understand the physiology of the ballistic motion of a body part, such as a limb across a joint. This translation of motion is characterized by the sequential contraction of the muscles executing the motion. The muscles across the joint contract in a timed-sequence from agonist to antagonist to agonist, expressed as the triphasic pattern agonist-antagonist-agonist. This precise cascade of events is carefully timed by the nervous system and when disrupted the phenomenon of dystonia arises [1].

Therefore, dystonia can be defined as a phasic (jerky) or sustained change (static) in the posture of a body part or segment, characterized by co-contraction of agonist and antagonist muscles, leading to altered geometry of the body part and impaired motor control. Despite its clinical motor phenotype, dystonia also involves impaired sensory processing and amelioration by the classic sensory trick, “la geste antagonistique”, or aggravation by high amplitude-high frequency vibrations. This latter aberration leads to the “overflow” of the disordered motor control into the neighboring muscles [2]. According to the Dystonia Medical Research Foundation (DMRF), dystonia is defined as excessive and repetitive muscle contractions that result in abnormal muscle movements and body posture that are stereotypic and may be painful and can interfere with motion control.

The first agonist burst generates the impulse force of motion followed by the antagonist burst which slows down the motion to the desired point. The agonist muscle reactivates to dampen any oscillations of motion. In dystonia, the duration of the agonist and antagonist bursts is prolonged, there is co-contraction of agonist and antagonist muscles and overflow of activity into the neighboring muscles. The peak velocity of the ballistic motion is also reduced. The timing, force and duration of the bursts is controlled by the striatum and the cerebellum [3].

The focal hand dystonias are a fascinating group of task-specific dystonias, that can involve penmanship (writer's cramp), playing the piano or guitar (musician's dystonia), or even swinging a golf club (the yips).

The prevailing theories of pathophysiology of focal hand dystonia center on the concept of disinhibition, overactivity of motor pathways, impaired sensory-motor integration and impaired motor plasticity. There is a plethora of data including functional magnetic resonance imaging (MRI) studies, electrophysiological studies (electromyography, somatosensory evoked potentials, motor-evoked potential, trans-cranial magnetic stimulation) [4]. These models focus on the role of the basal ganglia and sensory-motor cortex.

Recently, the role of the cerebellum has expanded. This is not surprising as the cerebellum is critical in timing the sequential activity of muscle groups and calibrating the force and velocity of limbs during the translation of motion [5].

We present a case of a focal hand dystonia, organic writer's cramp, with a dramatic improvement of drawing skills when the patient completed the task of drawing an Archimedes spiral on a vertical versus a horizontal surface. This variance between the horizontal and vertical plane we refer to as an “anisotropy”. It is the author's experience that this phenomenon is not infrequent in writer's cramp and has not been previously appreciated.

In order to understand cerebellar function one has to return to Pellionisz and Llinas’s spectacular tensorial theory of brain function, whereby the cerebellum is viewed as a time-space metric tensor. The central theme of their theory is that motor coordination should be defined as the transformation of a motor intention to a motor execution involving vectors in the frequency hyperspace of the brain [6].

We will heuristically prove that the anisotropy of spiral drawing in the perpendicular planes is due to a faulty cerebellar metric tensor or equivalently any part of the brain involved in the translation of motion. This is based on the idea that tensorial transformation is independent of the frame of reference, with the horizontal and vertical planes representing two different reference planes. If the cerebral (possibly cerebellar) metric tensor is not faulty, then the spiral drawing in the horizontal plane should match the drawing in the vertical plane. This is not the case in our patient with writer's cramp, where there is amelioration of drawing in the vertical plane compared to the horizontal plane.

## Case presentation

We present a right-handed 65-year-old man with a four-year history of handwriting difficulty. He struggles to write legibly and he is slow to complete a word, complaining that his hand is too shaky. He has maintained his ability to feed himself and carry out simple tasks for his job as a mechanic such as handling tools and utilizing keys. No head or hand tremors are reported with activities such as holding a newspaper or a utensil for eating. To improve his penmanship, he has learned sensory “tricks” through trial and error, and he has noted that his penmanship improves when he stabilizes his right elbow with his left hand.

Past medical history is significant for hypertension, for which he receives amlodipine 10 mg a day. Family history does not include an essential tremor or other forms of dystonia such as cervical dystonia or other movement disorder such as Parkinson's disease.

His gait was entirely normal with normal cadence and tandem-walk. His speech was fluent with normal phonation; volume and cadence. On cranial nerve examination, there was no evidence of blepharospasm, oromandibular dystonia or torticollis. Power was entirely preserved in the arms. Sequence motion of the fingers was entirely normal bilaterally with absence of a rest tremor, cogwheel rigidity and speed of motion was normal. Cerebellar examination showed normal co-ordination with absence of upper or lower extremity dysmetria and no intention tremor or rebound phenomenon. Romberg sign was absent and deep tendon reflexes were symmetric and lively.

**Figure 1:**
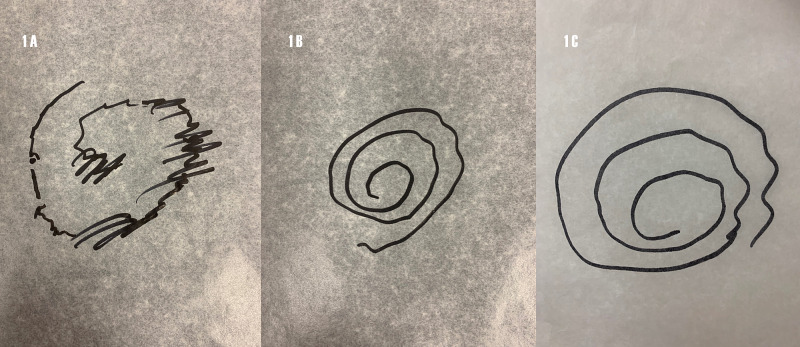
Spiral drawing. (A) Broken, ratchety spiral on a horizontal surface. (B) Smoother spiral on a vertical surface. (C) With the left hand holding the right elbow on a horizontal surface.

While outlining an Archimedes spiral, his penmanship displayed tremendous difficulty. He was able to hold a pen with ease but when he started drawing he hyperextended his right wrist and grasped the pen tightly with his thumb and index finger. The motion was very jerky and slow and it took him at least ten seconds to outline one circle of the spiral. The difference in accuracy was striking between drawing on a horizontal plane versus drawing on a vertical plane, with improvement also noted with the sensory trick outlined above (Figure 1).

A magnetic resonance imaging (MRI) study of the brain was normal. The patient declined forearm intramuscular botulinum toxin injections to improve his penmanship, citing that this deficit can be bettered by deploying his sensory trick and writing on a vertical plane with a clipboard on a wall, a technique we introduced to him.

## Discussion

We will sketch some of the basic ideas of the phenomenon of dystonia. We will discuss the pathophysiology of disinhibition, the role of the basal ganglia (namely the striatum), the sensory-motor cortex and the cerebellum. We will then discuss the evolution of concepts pertaining to the action of the nervous system, the organizing principles, from the simple reflex to tensor network theory. Finally we dive into the concept of tensors, which is challenging as one needs to be versed with relatively advanced mathematics. However, we will unravel the mathematics in quite simple language so that one can follow the arguments.

Firstly, the concept of surround-inhibition or lateral inhibition involves the suppression of an unwanted movement when a desired motor action is selected. In the sensory system this is akin to center-surround inhibition when enhancing the fidelity of a stimulus. Secondly, the basal ganglia are divided into the striatum, which is composed of the caudate nucleus and putamen, and the globus pallidus, which is composed of the globus pallidus externa (GPe) and the globus pallidus interna (GPi). The basal ganglia receive input from the ventrolateral thalamus and project to the motor cortex. There are two outflow pathways, the direct and indirect pathways:

The facilitatory direct pathway: Striatum $\rightarrow$ GPi $\rightarrow$Thalamus

The inhibitory indirect pathway: Striatum $\rightarrow$GPe $\rightarrow$STN $\rightarrow$ GPi $\rightarrow$ Thalamus

**Figure 2:**
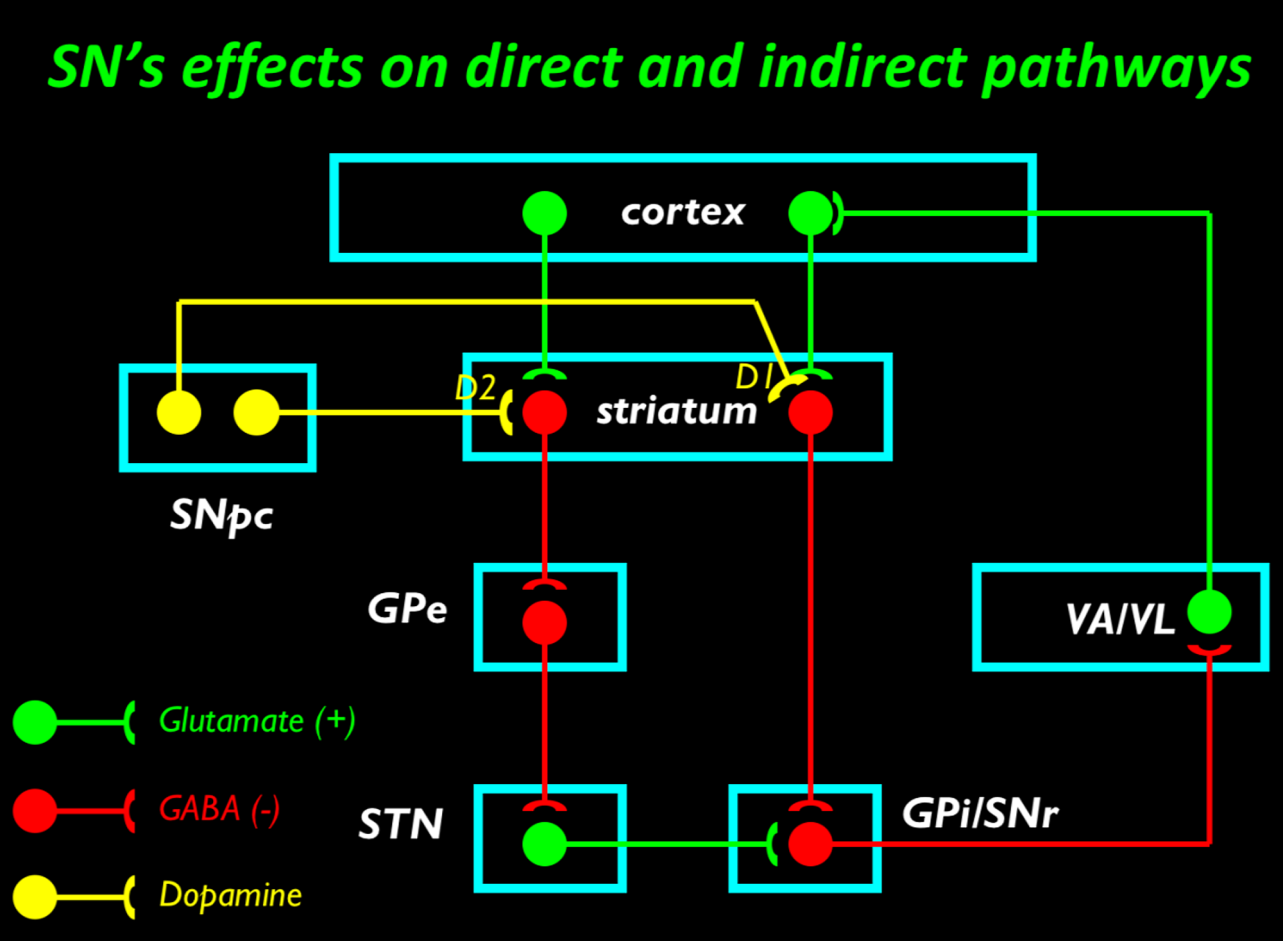
The direct and indirect pathways from the striatum to the cortex via the thalamus. Globus pallidus interna (GPi), Globus pallidus externa (GPe), Substantia nigra reticularis (SNr), Substantia nigra pars compacta (SNpc), Sub-thalamic nucleus (STN), gamma aminobutyric acid (GABA), ventral-anterior thalamus (VA), ventro-lateral thalamus (VL), excitatory (+), inhibitory (-).

The direct and indirect pathways are displayed below (Figure 2).

An overactive direct pathway or hypoactive indirect pathway can lead to undesired movement. The main mechanisms of disinhibition or hyperactivity of the motor pathways and of sensory expansion of the receptive fields are outlined below (Table 1) [7,8].

**Table 1:** Mechanisms of dystonia: the key idea is disinhibition of cortical and spinal cord circuits leading to motor hyperactivity and sensory expansion. Gamma hydroxybutyrate acid (GABA), transcranial magnetic stimulation (TMS), dopamine (D), negativity (N), somatosensory evoked potential (SSEP).

MECHANISM	LOCATION	PHYSIOLOGY	COMMENTS	MANIFESTATIONS
Lack of inhibition	Cortical, brainstem or spinal cord	Reduced reciprocal and surround inhibition / Shorter duration pause following motor-evoked potential	Loss of GABA-A inhibitory inter-neurons with intra-cortical inhibition. --GABA-B responsible for shorter pause following TMS. Reduced dopamine D2 receptor in putamen. Reduced GABA in motor cortex and basal ganglia	Unwanted movement and overflow. Lack of reciprocal inhibition leads to co-contraction of agonist and antagonist.
Abnormal motor preparation	Premotor cortex	Absent or reduced Bereitschaft Potential.	Cerebral preparation for voluntary action begins one to two seconds prior to the onset of movement and is bilateral. Two components: early negative component generated by supplementary motor area and cingulate motor area, and late negative component generated by the primary motor cortex.	Reduced peak velocity may be due to under-activation of first agonist burst
Abnormal sensory processing	Sensory cortex	Expansion of sensory receptive fields and abnormal sensory gating. Enlarged N30 with SSEP	The sensory representation of fingers is closer to each other in the sensory cortex with vibratory stimulation, as measured with positron-emission tomography.	Sensory trick and amelioration . Vibration Aggravates dystonia
Maladaptive plasticity	Cortex	Loss of surround inhibition	Low frequency peripheral nerve activation paired with supra-threshold trans-cranial magnetic stimulation leads to enhanced motor-evoked potentials, with reduced threshold of motor activation. Due to reduced intra-cortical inhibition and increased excitability of corticospinal tracts.	
Sensori-motor integration	Cortex	Reduced contingent negative variation	Referred to as Sensory-Motor Integrative Loop for Enacting (SMILE). This involves integrating movement preparation, sensory encoding and motor command that executes motion.	Abnormal motion fidelity: amplitude and timing of sequence of motion

One has to think of focal hand dystonia and other dystonias not just as basal ganglia disorders, a pre-motor disorder or a sensory-motor disorder but as a “network disorder”.

With respect to the “sensory trick”, the muscle spindles are activated (proprioception) and these afferent volleys travel in the dorsal spino-cerebellar tracts into the cerebellum. The muscle spindles can also be activated by high-frequency, high amplitude tonic vibrations, the aptly named tonic vibration reflex (TVR). When activated, this reflex worsens the tonic contractions. This effect is blocked by intramuscular injection of anesthetic [9].

The striatum has two compartments. The classical matrix compartment (calbindin-positive) consisting of the direct and indirect pathways, the classical “push-pull” model, and the striosomal pathway (tyrosine-hydroxylase-positive) involved in a nigro-striatal-nigro loop; the nigro meaning the substantia nigra. The latter pathway inhibits the direct facilitatory pathway.

Direct evidence of striatal pathology is provided by two rare genetic dystonic diseases (Table 2) [10].

**Table 2:** Genetic diseases highlighting the compartmentalization of the striatum into the striosome and matrix. Dystonia gene (DYT), dihydroxyphenylalanine (DOPA), guanosine triphosphate (GTP).

	X-LINKED DYSTONIA PARKINSONISM	DOPA RESPONSIVE DYSTONIA
GENE	DYT3	DYT5 (GTP cyclohydoxylase 1)
EPIDEMIOLOGY	Panay island / Philippines Onset - mid 30's	Childhood onset; females more than males
CLINICAL FEATURES	Focal dystonia, then Parkinsonism	Foot dystonia, then Parkinsonism, diurnal variation
PATHOLOGY	Dorsal striatum atrophy / loss of striosomal neurons	Loss of striosomal neurons
NETWORK DYSFUNCTION	Loss of striosomal inhibition of nigral neurons leading to direct pathway over-activity (via possible loss of GABA inhibition)	Loss of striosomal inhibition of nigral neurons leading to direct pathway over-activity (via loss of GABA inhibition)

A role for the cerebellum is provided by the observation that focal dystonia has been frequently described in the spino-cerebellar ataxias (SCA) and may modulate the progression of the ataxia, with greater severity in SCA 1, 2 and 3 and slower progression in SCA 6 [11]. There is also a cerebello-intrathalamic nucleus-striatal di-synaptic pathway that activates striatal dopaminergic neurons via cholinergic interneurons. This pathway triggers short-latency potentials that enhance synaptic plasticity when there is concurrent cortical stimulation [12,13]. We also outlined the role of the cerebellum in the duration of the agonist and antagonist bursts, and their sequential timing in the Introduction [3]. The findings on the role of the cerebellum has expanded over time with cerebellar neuronal dysfunction noted in rodent models of dystonia and with positron-emission tomography (PET) imaging and functional magnetic resonance imaging (fMRI) data implicating cerebellar pathways [14].

Having set the stage with the physiology, pathology and anatomy, we will next dive into the mathematics. Mathematics is a relation among numbers or variables, just as neurology is a relation among neurons. Understanding is a relation among facts just as geometry is a relation among points. Relations in the external world need to be synchronous with internal cerebral geometrical representations. For abstraction in geometry, the language we use is tensor calculus. The abstracting tool, tensor calculus, has to be able to mine the data, just like the proper tool to insert a nail into a ply of wood is a hammer and not one's hand. Abstraction has to relate structure to function. From the simple mono-synaptic deep-tendon reflex to Boolean algebra to differential geometry, function is correlated to structure via organizing principles.

The organizing principles of brain function are listed chronologically below as they unfolded over the last century (Table 3).

**Table 3:** Evolution of ideas regarding organizing principles of brain function.

SOURCE	ORGANIZING PRINCIPLE	LIMITATIONS
Sherrington, 1906 [15]	Reffexes	Too simple
McCulloch and Pitts, 1943 [16]	Logical operations	The sensori-motor paradigm is not Boolean
Robinson, 1968 [17]	Linear system theory	Does not account for non-linear systems, which predominate
Raibert, 1978 [18]	A library of pre-determined programs	Not true
Poggio and Reichardt, 1981 [19]	Non-linear systems theory	Incomplete; relies on predictive theory of Taylor expansion
Pellionisz and Llinas, 1979 [20]	Tensor calculus	Unclear if vector space is Euclidean or Non-Euclidean

We introduce the basic concepts of tensor calculus for the mathematically-seasoned (Appendix I). For the layman, we introduce the idea of a covariant and contravariant vectors or tensors. In very simple terms, we can think of a covariant vector as a gradient, expressed mathematically as

$$\frac{\partial }{\partial x^{\nu }}=\partial _{\nu }$$

and the contravariant vector as a position vector, expressed mathematically as

$$x^{\nu }$$

We can go back and forth between the covariant and contravariant tensor by the use of a metric tensor. The power and beauty of metric tensors is that they are coordinate-invariant. This means that distances measured in one frame of reference are identical to those measured in another frame of reference.

The work of Pellionisz and Llinas has shown that the cerebellum can be modeled as a metric tensor. The covariant and contravariant vectors can be thought of as multi-dimensional frequency vectors, with each individual neuron represented by its frequency of discharge. The covariant vector is constructed as a pre-image of the desired movement. It is a crude analogue, a simulacrum. This covariant vector needs to be transformed by the cerebellum, the metric tensor, into a contravariant vector, the actual and desired motion. Failure of the metric tensor, for instance the cerebellum, to exact this transformation can lead to faulty motion, since the covariant vector is a crude copy of the intended motion. We need the covariant vector to be transformed to a contravariant tensor for accuracy of motion. This hypothesis has proven to be an excellent model for the vestibulo-ocular reflex with replication of experimental data [20]. The strength of tensors lies in their ability to incorporate complexity. For example, a rank four tensor can yield 256 equations, the rank of a tensor being the number of indices that it carries. The mathematical machinery of tensor theory is ideally suited and can handle the incredibly complexity of the human brain, especially the number of neural connections, which may be embedded in a central nervous system frequency hyperspace (Appendix II).

In our case, the fact that our patient’s horizontal-plane spiral drawing was crude compared to the drawing on the vertical-plane implies that there is a faulty metric tensor somewhere in the nervous system, likely the cerebellum. Paraphrasing, there is variance or lack of invariance of coordinate transformation, a central tenet of tensor transformation, noting that tensor transformations should preserve distance across coordinate systems or reference frames.

Future studies should be directed at studying functional MRI and PET studies of patients performing tasks not just in the perpendicular planes (horizontal and vertical) but at other various planar angles.

## Conclusions

The dystonias are a fascinating group of disorders with quite a unique and conspicuous presentation. Their pathophysiology is quite intriguing and continually being updated. The classical notion of dystonia being solely a basal ganglia disorder is no longer tenable and has now expanded to include the cerebellum and the sensory-motor cortices. In fact, one has to think of dystonia as a disorder of network function. Very few theories are better armed to handle the complex networks of the brain than the tensor theory of vectors. For motor action, the brain has to transform an internal representation of the desired action into an accurate efference copy. The huge advantage of tensors is the fact that they preserve distances between reference frames, as in the case of the external world and the internal representation of that world. Our case of focal hand dystonia demonstrates an unequivocal breakdown of the tensorial networks of the brain with variance of images in two different planes. This is a novel pathophysiology for focal hand dystonias, and may hold true in general for other dystonias, as the different forms of dystonia parallel each other pathophysiologically.

## Appendices

$I$ Basic introduction to tensors

Starting with a coordinate system $x^{'\mu }=x^{'\mu }(x)$, and a scalar field $\varphi (x)$, we deploy the Einstein summation convention and define a covariant tensor with a subscript, according to the transformation law

$$\frac{\partial \varphi }{\partial x^{'\mu }}=\frac{\partial \varphi }{\partial x^{\nu }}\frac{\partial x^{\nu }}{\partial x^{'\mu }}$$

By the same token, any vector $A_{\nu }(x^{'})$, that follow this transformation law

$$A_{\nu }(x^{'})= \frac{\partial x^{\nu }}{\partial x^{'\mu }}B_{\nu }(x)$$

is a covariant tensor.

A contravariant tensor, represented by a superscript, follows a different transformation law

$$dx^{'\mu }(x^{'})=\frac{\partial x'^{\mu }}{\partial x^{\nu }}dx^{\nu }$$

and by similar reasoning, any vector $A^{'\mu }(x^{'})$ that follow the same transformation law

$$A^{'\mu }(x^{'})=\frac{\partial x'^{\mu } }{\partial x^{\nu } }B^{\nu }(x)$$

is a contravariant tensor.

Note the reciprocal relationship between the covariant and contravariant tensors.

We demand that,

$$\frac{\partial x^{'\mu }}{\partial x'^{\nu } }=\frac{\partial x^{\mu }}{\partial x^{\nu }}=\delta {_{\mu }}^{\nu }$$

the Kronecker delta, $\delta {_{\mu }}^{\nu }=1, \nu =\mu ;$ and $\delta {_{\mu }}^{\nu }=0, \nu \neq \mu ;$.

The invariance of tensors is a gift from heaven. If we take the scalar product of a covariant and contravariant tensor

$$\frac{\partial x^{\nu }}{\partial x^{'\mu }}A_{\nu }\frac{\partial x^{'\mu }}{\partial x^{\alpha }}B^{\alpha }$$

$$A^{'}B^{'}= \frac{\partial x^{\nu }}{\partial x^{'\mu }}A_{\nu }\frac{\partial x^{'\mu }}{\partial x^{\alpha }}B^{\alpha }=\frac{\partial x^{\nu }}{\partial x^{\alpha }}A_{\nu }B^{\alpha }=\delta {_{\alpha }}^{\nu }A_{\nu }B^{\alpha }=A_{\alpha }B^{\alpha }=AB$$

Therefore, the scalar product is preserved in both reference frames.

$II$ The metric tensor

The metric tensor is a summation of the basis vectors

$g_{ij} = e_{i}.e_{j}$

The line element is

$$ds^{2}= g_{ij}dx^{i}dx^{j}$$

The metric and inverse metric are related by the Kronecker delta

$$g_{ij}g^{jl}=\delta {_{i}}^{l}$$

The metric tensor can be used to raise and lower indices; transforming covariant to contravariant vectors and vice versa

$$A^{i}=g^{ij}A_{j}$$

and

$$A_{i}=g_{ij}A^{j}$$

Taking the scalar product of a covariant and a contravariant vector

$$A_{i}g^{ij}B^{j}g_{ij}= A_{i}B^{j}\delta {_{i}}^{j}=A_{i}B^{j}=A.B$$
